# Structural Characterization and Immune Activation Capacity of Peptidoglycan from *Corynebacterium glutamicum* in RAW264.7 Cells

**DOI:** 10.3390/ijms26010237

**Published:** 2024-12-30

**Authors:** Xiaoying Wang, Shuzhen Li, Aijuan Zheng, Zhimin Chen, Jiang Chen, Zhiheng Zou, Guohua Liu

**Affiliations:** 1 Key Laboratory of Feed Biotechnology of the Ministry of Agriculture and Rural Affairs, Risk Assessment Laboratory of Animal Product Quality Safety Feed Source Factors of the Ministry of Agriculture and Rural Affairs, Institute of Feed Research of Chinese Academy of Agricultural Sciences, Beijing 100081, China; 2 Institute of Animal Husbandry and Veterinary Science, Jiangxi Academy of Agricultural Sciences, Nanchang 330200, China

**Keywords:** *Corynebacterium glutamicum*, peptidoglycan, immune activation, RAW264.7 cells, cytokines

## Abstract

Peptidoglycan (PGN) is a unique component of prokaryotic cell walls with immune-enhancing capacities. Here, we extracted PGN from *Corynebacterium glutamicum*, a by-product of amino acid fermentation, using the trichloroacetic acid (TCA) method. SDS-PAGE analysis confirmed the presence of PGN, with a band of approximately 28 kDa. Further analysis was conducted through amino acid analysis, FTIR, and MALDI-TOF/TOF MS, and the results showed that the chemical structural monomer of PGN is NAG-(β-1,4-)-NAM-l-Ala-d-Glu-l-Lis-d-Ala. The immune activation effects of PGN were evaluated in a RAW264.7 cell model. Our results showed that PGN could increase the secretion level of NO, ROS, and immune regulatory substances, including TNF-α and IL-1β, and up-regulated the mRNA expression of *TNF-α* and *iNOS*. In addition, PGN stimulated the expression of *ERK2*, *MyD88*, *RIP2*, and the related receptor *NOD1* in the NF-κB and MAPK pathways. Comparative RNA sequencing was conducted to analyze the gene expression profiles in RAW264.7 cells. KEGG analysis indicated that most of the genes were enriched in the NF-κB, MAPK, and TNF signaling pathways. Taken together, these findings suggest that PGN may have immune-activating potential for the development and application of immune adjuvants. Importantly, the application of PGN also provides a new way to utilize amino acid fermentation by-products.

## 1. Introduction

Adjuvants are substances that can enhance the immune response to vaccines. Currently available adjuvants include aluminum salt formulations, oil emulsions, and bacterial derivatives [1,2], which have the ability to induce and enhance specific adaptive immune responses by targeting innate immune cells and activating pattern recognition receptor (PRR) signaling pathways [3,4]. Many plant polysaccharides have been reported as vaccine adjuvant candidates due to their wide range of sources and, in particular, their nutritional and therapeutic potential, which has been proven [5,6,7,8,9]. Polysaccharides of microbial cell wall origin, such as peptidoglycan (PGN), have been studied as immune adjuvants to initiate both non-specific and specific immune responses [10,11].

PGN, a unique component of prokaryotic cell walls, is a polymer composed of disaccharide units and peptides. The basic skeleton of PGN consists of *N*-acetylmuramic acid (NAM) and *N*-acetylglucosamine (NAG), which are connected by a β-1,4 glycosidic bond. As a bacterial skeleton, PGN has the function of protecting cells from osmotic pressure and maintaining the normal morphology of cells [12,13]. The tetrapeptide chain of PGN is connected via the 3-O-lactam group of N-acetylmuramic acid, with the amino acid molecules on the chain are alternately connected in L-type and D-type configurations [12]. The typical linkage sequence in PGN consists of L-alanine, D-glutamic acid, L-lysine or endo-diaminoheptanedioic acid, and D-alanine [12,14,15], but this sequence may vary among different bacterial strains. The fundamental structure of PGN disaccharide units is consistent across different bacteria, with variations primarily found in the amino acid composition of the peptide chains and the method of cross-linking between these chains [12]. In Gram-negative bacteria, a majority of the peptide chains from two peptidoglycan molecules are directly linked, constituting about 50% of all peptide chains. Conversely, in Gram-positive bacteria, cross-linking occurs via a network of peptide bridges [12]. PGN is predominantly found in both Gram-positive and Gram-negative bacteria [16]. In Gram-positive bacteria, PGN typically forms a multilayered macromolecule, representing 50–95% of the cell wall’s dry weight, while in Gram-negative bacteria, it primarily forms a monolayer, constituting 5–20% of the cell wall’s dry weight [12,14].

PGN exhibits various biological activities, including immunomodulation, anti-infection, anti-tumor effects, cytotoxicity, and adhesion. Eukaryotes do not contain PGN, but they contain PGN recognition proteins (PGRPs) that enable them to detect invading bacteria and initiate non-specific immune responses [17]. Therefore, PGN becomes one of the most ideal targets for the eukaryotic immune system to enhance non-specific systemic immunity. When used as an adjuvant in vaccines or independently, PGN can increase the expression of cytokines in several immune cells and enhance antibody-dependent specific responses [18,19]. For instance, when mice were injected intraperitoneally with PGN, significant increases in the levels of nitric oxide (NO), interleukin-6 (IL-6), interleukin-12 (IL-12), and tumor necrosis factor-alpha (TNF-α) were observed in peritoneal macrophages [20]. In addition, mixing PGN monomers with incomplete Freund’s adjuvant and immunizing NIH/OlaHsd mice with ovalbumin as an antigen induced a shift toward an ovalbumin (OVA)-specific Th2-type immune response [21].

*Corynebacterium glutamicum* was initially identified by Kinoshita in 1957 as a glutamate-producing bacterium, and since then, it has been the primary organism used globally in the amino acid fermentation industry for over four decades [22]. Monosodium glutamate (MSG) is a kind of sodium salt of L-glutamic acid, and as the major producer, China produces millions of tons of MSG annually, along with a substantial amount of by-products [23]. Traditionally, waste bacterial sludge has been utilized primarily for the extraction of glutamate thalli protein, but there is a pressing need to explore and optimize its further development and utilization potential.

Furthermore, PGN varies structurally across bacterial species, and the specific roles and mechanisms by which PGNs from different bacteria serve as immune adjuvants remain incompletely understood. In this study, we extracted PGN from *Corynebacterium glutamicum* and investigated its immune activation capacities in a cell model. Additionally, we analyzed the inflammatory regulatory signaling pathways and explored the potential mechanisms through which PGN functions as an immune adjuvant.

## 2. Results

### 2.1. Extraction and Structural Analysis of PGN

The extraction rate was calculated based on the ratio of the dry matter of the extract to the wet weight of the bacteria, and the extraction rate of PGN based on the TCA method was 18.79%. The contents of protein, total carbohydrates, hexosamine, and fat in PGN from *Corynebacterium glutamicum* were 33.54%, 25.62%, 29.94%, and 1.9%, respectively (Table 1).

**Figure 1:**
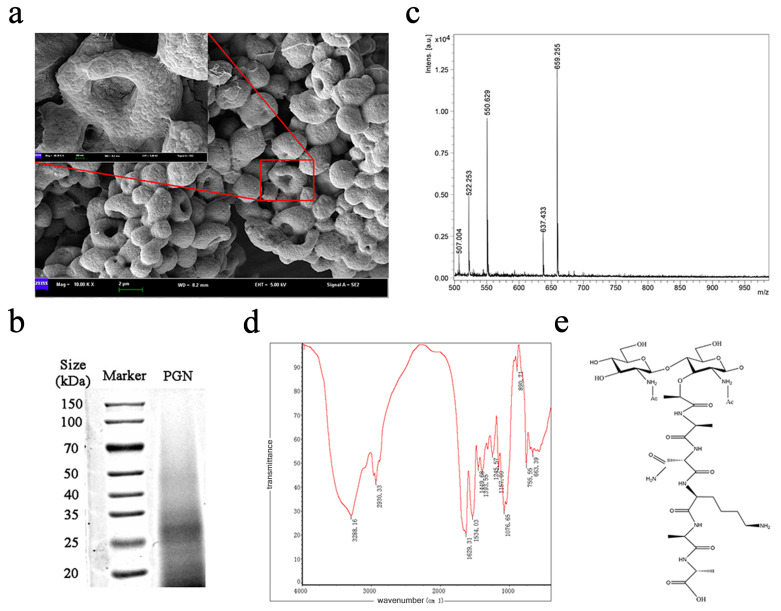
Chemical structure of PGN from *Corynebacterium glutamicum*. (**a**) Examination via SEM at original magnification (1000×) and 5000×. (**b**) SDS-PAGE analysis of PGN proteins. (**c**) MALDI-TOF/TOF MS analysis of PGN. (**d**) FTIR analysis of PGN. (**e**) Chemical structure of PGN.

As shown in Figure 1a, the SEM images reveal that the extracted PGN maintains the same structure as the bacterium, appearing as tightly connected spheres with a concave center. In this study, we extracted the intact peptidoglycan from *Corynebacterium glutamicum* using a non-physical fragmentation method, which preserved its morphological structure. SDS-PAGE protein electrophoresis was used to determine the molecular weight of PGN. The bands faded due to the low solubility of PGN, but their locations can be clearly seen in Figure 1b. This indicates that the molecular weight of the extracted PGN was relatively uniform, with a band molecular weight of approximately 28 kDa. The amino acid composition analysis results showed that the main amino acid components were glutamic acid, aspartic acid, leucine, lysine, and histidine, among others (Table 2). The infrared absorption spectra of PGN were determined using the KBr compression method. We found that the extracted PGN exhibited the characteristic absorption peaks of peptidoglycan, as shown in Figure 1c. The major mass-to-charge (*m*/*z*) ratios of PGN from *Corynebacterium glutamicum* were 507.004, 522.253, and 550.629.

### 2.2. Effect of PGN on Viability in RAW264.7 Cells

**Figure 2:**
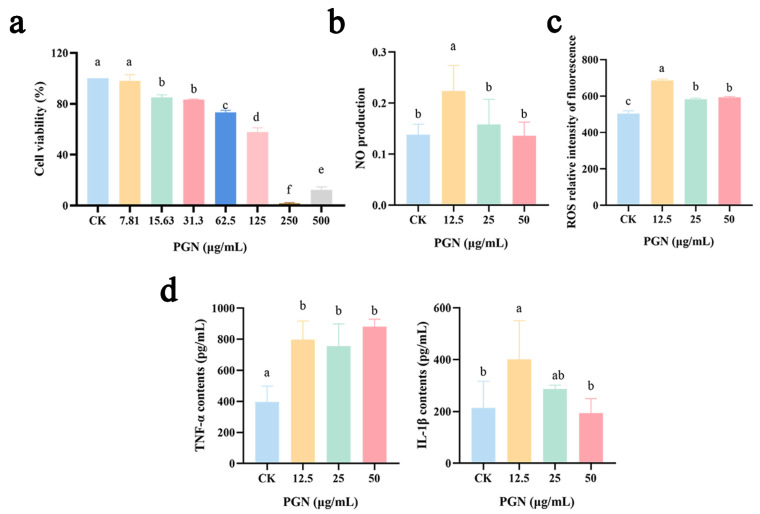
Effects of different PGN concentrations on the immune-active substances in RAW264.7 cells. (**a**) Cell viability. (**b**) NO production. (**c**) ROS release. (**d**) TNF-α and IL-1β secretion. PGN refers to peptidoglycan extract. PGN concentrations of 0, 12.5, 25, and 50 μg/mL were used as control (CK), low, medium, and high PGN groups, respectively. Data are presented as mean ± SD from three independent experiments. Values indicated with different letters differ significantly (*p* < 0.05) compared to the control (CK) group, as determined by one-way ANOVA.

To study the dose effect of PGN on cells, the viability of RAW264.7 cells was detected using the CCK8 method after incubation with different levels of PGN for 24 h. Our results showed that the viability of RAW264.7 cells decreased with the increase in PGN concentration (Figure 2a). The half maximal inhibitory concentration (IC50) value was calculated to be 110.1 μg/mL using SPSS. Therefore, subsequent PGN concentrations of 0, 12.5, 25, and 50 μg/mL were used as control (CK), low, medium, and high PGN dose groups, respectively.

### 2.3. Effect of PGN on NO Production in RAW264.7 Cells

A standard curve was constructed by analyzing the relationship between different concentrations of standard NaNO_2_ and the corresponding OD540 values. Linear correlation and regression analyses were performed, yielding the following linear regression equation: Y = 0.0117X + 0.0479, R^2^ = 0.999. There was a certain correlation between the standard NaNO2 content and the corresponding OD value. By substituting the OD values of different samples into the linear equation, the corresponding NO production was calculated. The results showed that RAW264.7 cells incubated with different concentrations of PGN for 12 h exhibited a significant dose-dependent increase in NO production compared to the CK group (*p* < 0.05) (Figure 2b).

### 2.4. Effect of PGN on Reactive Oxygen Species (ROS) Release in RAW264.7 Cells

Figure 2c shows the release of ROS from RAW264.7 cells treated with different concentrations of PGN. All treatments with different concentrations of PGN significantly increased ROS release compared to that in the CK group (*p* < 0.05). Notably, concentration, the release of ROS was the highest in the low-PGN-concentration group.

### 2.5. Effect of PGN on Cellular IL-1β and TNF-α Secretion in RAW264.7 Cells

Figure 2d shows that different concentrations of PGN significantly enhanced TNF-α secretion compared with that in the CK group (*p* < 0.05). Additionally, IL-1β secretion was significantly increased in the low-PGN-concentration group (*p* < 0.05), while in the medium-dose group, PGN was able to increase the secretion of IL-1β, but not significantly (*p* > 0.05).

### 2.6. Effects of PGN on IL-1β, Interleukin-6 (IL-6), and TNF-α Expression in RAW264.7 Cells

**Figure 3:**
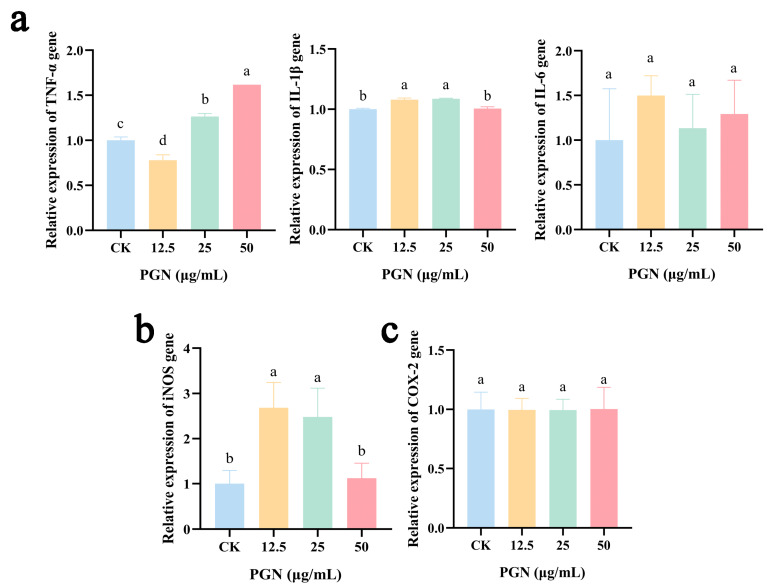
Effects of different concentrations of PGN on the gene expression of immune-active substances in RAW264.7 cells. (**a**) *TNF-α*, *IL-1β*, and *IL-6*. (**b**) *iNOS*. (**c**) *COX-2*. PGN refers to peptidoglycan extract. PGN concentrations of 0, 12.5, 25, and 50 μg/mL were used as control (CK), low, medium, and high PGN groups, respectively. Data are presented as mean ± SD from three independent experiments. Values indicated with different letters differ significantly (*p* < 0.05) from the control (CK) group, as determined by one-way ANOVA.

As shown in Figure 3a, the treatment of RAW264.7 cells with PGN led to a significant decrease in *TNF-α* expression in the low-dose group, while a significant increase was observed in the medium- and high-dose groups compared with values in the CK group (*p* < 0.05). The expression of *IL-1β* was significantly elevated both in the low- and medium-PGN-level groups (*p* < 0.05), whereas the expression of *IL-6* was not affected (*p* > 0.05) in any PGN treatment groups compared with that in the CK group.

### 2.7. Effects of PGN on Inducible Nitric Oxide Synthase (iNOS) and Cyclooxygenase-2 (COX-2) Expression in RAW264.7 Cells

The cytokine induction of iNOS leads to the production of NO. We performed RT-qPCR analysis to investigate the role of PGN in the expression of *iNOS* and *COX-2*. The result showed that *iNOS* expression was dose dependent, with a significant increase in the low- and medium-PGN-level groups (*p* < 0.05), as shown in Figure 3b. However, there was no effect on *COX-2* expression (*p* > 0.05), as depicted in Figure 3c.

### 2.8. Effects of PGN on Related Signaling Pathway Gene Expression in RAW264.7 Cells

**Figure 4:**
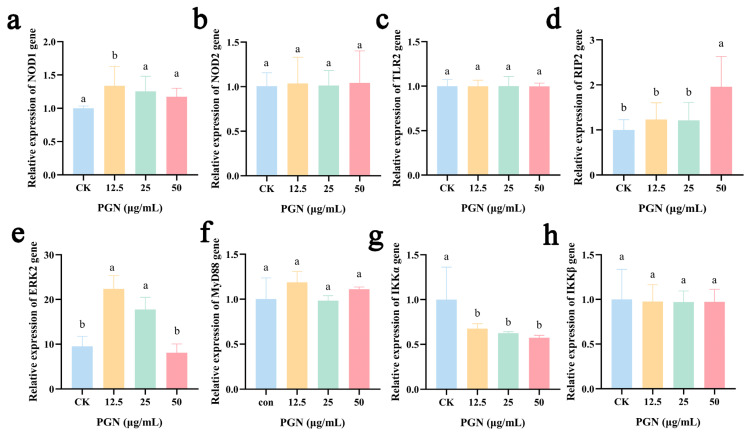
Effects of PGN on the expression of related signaling pathway genes in RAW264.7 cells. (**a**) *NOD1*. (**b**) *NOD2*. (**c**) *TRL2*. (**d**) *RIP2*. (**e**) *ERK2*. (**f**) *MyD88*. (**g**) *IKKα*. (**h**) *IKKβ*. PGN refers to peptidoglycan extract. PGN concentrations of 0, 12.5, 25, and 50 μg/mL were used as control (CK), low, medium, high PGN groups, respectively. Data are presented as mean ± SD from three independent experiments. Values indicated with different letters differ significantly (*p* < 0.05) compared to the control (CK) group, as determined by one-way ANOVA.

To investigate the effects of PGN on the expression of specific pathway genes in RAW264.7 cells, we performed RT-qPCR analyses (Figure 4). We found that PGN significantly increased the expression of *nucleotide binding oligomerization domain containing 1* (*NOD1*) in the low-PGN-dose group (Figure 4a), *receptor interacting protein-2* (*RIP2*) in the high-PGN group (Figure 4d), and *ERK2* in both low- and medium-dose groups (Figure 4e) (*p* < 0.05). Interestingly, the expression of *IKK alpha* (*IKKα*) was reduced at different concentrations (Figure 4g), with statistical significance (*p* < 0.05).

### 2.9. Transcriptome Changes in Gene Expression in RAW264.7 Cells by PGN

**Figure 5:**
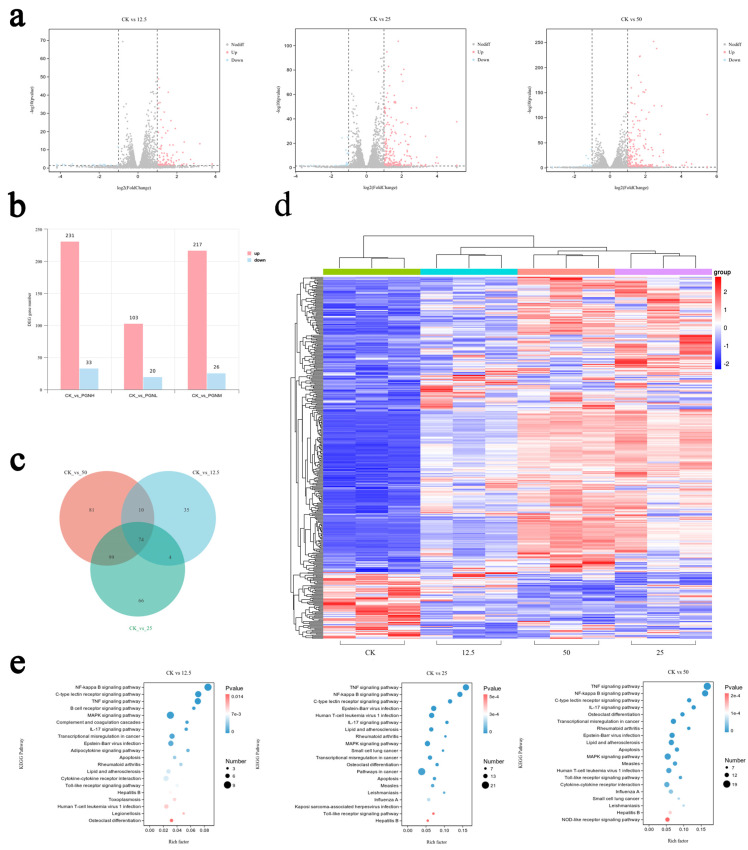
Transcriptomic changes in gene expression in RAW264.7 cells by PGN. (**a**) Volcano plot of mRNA expression in CK and low-, medium-, and high-PGN-dose groups. (**b**) Identification of DEGs (differentially expressed genes) by RNA sequencing. (**c**) Venn diagram analysis of differentially expressed mRNAs among the three groups. (**d**) Heat map of differentially expressed genes showing stratified clustering of the four groups. (**e**) KEGG top 20 enrichment of differentially expressed mRNAs between the CK and the three PGN groups.

To investigate the transcriptomic changes in gene expression in RAW264.7 cells induced by PGN, we performed differentially expressed gene (DEG) analysis using RNA-seq data. Differential gene expression across PGN-treated groups (low-, medium-, high-dose) compared with that in the CK group is shown in a volcano plot (Figure 5a). DEGs were identified as up- or down-regulated and are shown as bars (Figure 5b). We listed the number of differentially regulated genes in the CK and PGN-treated groups. As shown in Figure 5c, the overlapping arcs show the number of genes shared between these three categories. There were more DEGs between the CK and high-dose (CK vs. H) and the CK and medium-dose (CK vs. M) groups than between the CK and low-dose groups (CK vs. L). In addition, 241 genes associated with changes relative to the CK group were selected, and their expression profiles were analyzed by group comparison (Figure 5d). To further find the relevant biological pathways, KEGG enrichment analysis of DEGs was performed using the annotation data. The most significantly enriched pathways were the nuclear factor-κB (NF-κB), mitogen-activated protein kinase (MAPK), and TNF signaling pathways. The top 20 KEGG pathways are shown in Figure 5e.

## 3. Discussion

The major component of most bacterial cell walls is PGN, which is mainly composed of disaccharide units and peptides [13]. In the present study, the total carbohydrate content and protein content of PGN were high, at 33.54% and 29.94%, respectively. This also directly confirms that the structural composition of PGN monomer is a disaccharide-linked polypeptide. This was consistent with research by Wu [24]. Furthermore, the determination of hexosamine content also revealed that the PGN monosaccharide structure contains N-acetylglucosamine (NAG), which is consistent with the finding that the disaccharide unit of peptidoglycan consists of NAM and NAG [12]. Thus, the primary components of the isolated PGN are glycans and proteins.

FTIR analysis showed that the N-H bending vibration was located at 755.55 cm^−1^, the carbonyl C=O stretching vibration peak at 1076.65 cm^−1^, and the amide II region at 1534.09 cm^−1^, indicating the presence of substituted tetrapeptide stems in the NAM residues. The amide I region was located at 1629.31 cm^−1^, indicating the presence of an amide bond functional group in the peptidoglycan. The methyl methylene C-H stretching vibration absorption peak, which appears at 2930.33 cm^−1^, represents the polysaccharide residue (probably NAG-(β-1,4)-NAM) in this sample. The findings were consistent with the established structure of peptidoglycan, confirming the presence of NAG residues in PGN and suggesting the likely presence of a NAG-(β-1,4)-NAM band [12].

To investigate the structure of *Corynebacterium glutamicum* PGN, we analyzed results related to MALDI-TOF/TOF MS analysis. The results showed that the molecular weights of the major PGN oligosaccharides were in the range of 500–700 *m*/*z*. However, it was not possible to confirm the accuracy of the structure of the PGN from this information. Nevertheless, the results from amino acid analysis and FITC staining offered additional insights into the structure of PGN.

Amino acid analysis is a crucial indicator for peptidoglycan characterization [25]. Our results showed that the main amino acids were glutamic acid, aspartic acid, leucine, lysine, histidine, etc. Additionally, the peptide chain consisted mainly of alanine, glutamic acid, lysine, and meso-heptanedioic acid [12]. This suggests that PGN from *Corynebacterium glutamicum* may be a Lys type.

The composition and morphology of PGN were determined by SEM and FTIR analyses, while MALDI-TOF/TOF MS and amino acid analysis provided strong evidence for its structure. The chemical structure monomer of PGN from *Corynebacterium glutamicum* is NAG-(β-1,4)-NAM-l-Ala-d-Glu-l-Lys-d-Ala (Figure 1e).

In our study, we confirmed that the PGN used has an immune activation capacity. Specifically, we observed an increase in the mRNA expression of iNOS, as well as an increase in NO production and ROS release, which is similar to the findings of previous research [20,26]. The activation of iNOS enables the intracellular synthesis of a range of nitrogen compounds such as NO. As a versatile signaling and effector molecule, NO possesses a wide array of biological functions. It is particularly crucial for the activation of macrophages, enabling them to eliminate tumor cells and pathogenic microorganisms [27]. Additionally, PGN treatment led to an increase in the levels of TNF-α and IL-1β following PGN treatment in RAW264.7 cells, aligning with the findings from RT-qPCR analysis. Cytokines, such as TNF-α and IL-1β, play a crucial role in regulating the immune response and mediating inflammation [28]. PGN has already been demonstrated to activate the immune system by stimulating mononuclear phagocytes and endothelial cells to release immune regulatory substances, including tumor necrosis factor-alpha (TNF-α), various interleukins (IL-1, IL-6, IL-8, IL-12), and interferon gamma (INF-γ), as well as ROS and lipids [29,30,31]. Thus, PGN is also expected to play an important role in enhancing host innate immunity.

PGN is one of the ideal targets recognized by the eukaryotic immune system to enhance non-specific systemic immunity, with many studies on the mechanisms by which PGN is recognized by target cells [31]. The NOD1 receptor is predominantly sensitive to gamma-D-glutamyl-meso-diaminopimelic acid (iE-DAP), a distinctive peptidoglycan fragment that is commonly found in the cell walls of both Gram-negative and certain Gram-positive bacteria. Meanwhile, NOD2 is primarily responsive to muramyl dipeptide (MDP), a compound composed of N-acetylmuramic acid and D-isoglutamine that is a key component of peptidoglycan in the cell walls of Gram-positive bacteria. However, possible cellular recognition mechanisms regarding the PGN of *Corynebacterium glutamicum* origin have not yet been investigated. In our study, we examined the expression of *NOD1*, *NOD2*, and *TLR2* receptors and found that the expression of *NOD1* was increased significantly. This finding was consistent with previous studies reporting the identification of PGN mechanisms [32]. It has been shown that NOD1, when activated, can interact with RIPK2 to activate the NF-κB and MAPK pathways, which leads to the production of inflammatory cytokines and chemokines [31,33]. Hence, we examined the expression of *RIP2*, *IKKα*, *IKKβ*, *MyD88*, and *ERK2*. We observed that PGN elevated the expression of *RIP2*, *MyD88*, and *ERK2*. This suggests that the present study is consistent with reported studies in which PGN activated the NOD1 receptor and bound to RIP2, subsequently activating the NF-κB and MAPK signaling pathways [27]. Additionally, we again performed transcriptomics analysis to assess how PGN treatment affects gene expression in RAW264.7 cells. The functional enrichment analysis of DEGs revealed pathways associated with the NF-κB, TNF, and MAPK signaling pathways, consistent with our initial findings. Therefore, the information on the cellular recognition mechanisms of PGN elucidated in our study will provide useful insights into the biological responses induced by PGN in the host. Future research should verify the immune activation effect and molecular mechanism of PGN from *Corynebacterium glutamicum* in vivo.

## 4. Materials and Methods

### 4.1. PGN Isolation

*Corynebacterium glutamicum* (CICC 10053) was purchased from the China Center of Industrial Culture Collection (https://www.china-cicc.org/), and an overnight culture of bacteria was inoculated into beef extract peptone broth (LA8790, Solarbio Science & Technology Co., Ltd., Beijing, China) and then cultured at 30 °C with gentle shaking until it reached an optical density of 1.5 at 600 nm.

The bacterial precipitate was centrifuged and then washed three times with saline. PGN was extracted using the TCA method, as described by Sekine [34]. The precipitate was then resuspended in 10% TCA (V/m = 10:1), boiled for 30 min in a water bath, and then centrifuged at 12,000 r/min for 15 min to collect the precipitate, which was subsequently washed three times with distilled water. The precipitate was added to a mixture of sodium acetate (pH 4.6), chloroform, and methanol (4:5:10 V/V/V) and allowed to stand for 24 h. After centrifugation at 8000 r/min for 20 min, the precipitate was transferred to Tris-HCl (0.1 M, pH 7.5) containing 1 mg/mL of trypsin and incubated at 37 °C with shaking at 120 r/min for 12 h. The precipitate was collected by centrifugation at 8000 r/min for 20 min, washed three times with distilled water, and finally lyophilized and stored in a refrigerator at 4 °C for further analysis.

### 4.2. Analysis of Protein, Total Carbohydrate, and Hexosamine Composition in PGN

The total carbohydrate content of PGN was determined using the sulfuric acid–phenol method [35]. Standard curves were plotted based on regression equations with regression coefficients ≥ 0.99 (Y = 0.0049X + 0.0603, R^2^ = 0.9958). The protein content was determined by the Bradford method [36]. Standard curves were plotted based on regression equations with regression coefficients ≥ 0.9958 (Y = 1.1015X + 0.3543, R^2^ = 0.9985). The content of N-acetylaminohexose was determined using the Morgan–Elson reaction [37]. Standard curves were plotted based on regression equations with regression coefficients ≥ 0.9958 (Y = 0.0065X, R^2^ = 0.9913). The fat content was determined using the vanillin method. Standard curves were plotted based on regression equations with regression coefficients ≥ 0.9916 (Y = 0.0008X + 0.0734, R^2^ = 0.9916).

### 4.3. Chemical Structure Analysis of PGN

PGN was immersed in 2% glutaraldehyde at 4 °C and then observed by scanning electron microscopy (SEM) (S-3400 N, Hitachi Corp., Tokyo, Japan).

The molecular weight of PGN was determined by SDS-PAGE analysis. Electrophoresis was conducted in a 30% polyacrylamide gel and then stained with Kaomas Brilliant Blue G250 (6104-58-1, Solarbio Science & Technology Co., Ltd., China).

Different methods for amino acid analysis have been developed and are commercially available. The amino acid composition of PGN was analyzed using a fully automated amino acid analyzer (Hitachi L-8900, Tokyo, Japan; Centre for Feed Testing and Safety Evaluation, Institute of Feed Research, Beijing, China).

MALDI-TOF/TOF MS and Fourier-transform infrared spectroscopy (FTIR) were used to determine the molecular weight and structural differences in PGN from *Corynebacterium glutamicum*. For MALDI-TOF/TOF MS, 1 µL of PGN was mixed with 1 μL of saturated 2,5-dihydroxybenzoic acid (DHB) matrix solution (50 mg/mL). A droplet (1 μL) of the resulting mixture was placed on the sample target and dried at room temperature. The sample was then loaded into the mass spectrometer and analyzed. FTIR spectroscopy in the relevant region (4000–400 cm^−1^) was also used for the structural analysis of PGN. FTIR analyses were carried out to examine the O-H absorption bands, C=O stretching vibrational bands, N-H deformation, and C-N stretching.

### 4.4. Cell Culture

Mouse macrophage cells (RAW264.7) were provided by our laboratory and cultured in Dulbecco’s modified Eagle’s medium (DMEM) with high glucose and L-glutamine (Gibco, Grand Island, NY, USA) containing 10% (*v*/*v*) endotoxin-free fetal bovine serum (FBS), (11011-861, Zhejiang Tianhang Biotechnology Co., Ltd., Tongxiang, China), 1% penicillin (5000 U/mL), and 1% streptomycin (5000 U/mL) (Gibco, Grand Island, NY, USA) at 37 °C, 5% CO_2_ atmosphere.

### 4.5. Cell Viability Test

Cell viability was assessed with a Cell Counting Kit-8 (C0038, Beyotime Biotech Inc., Haimen, China) assay. RAW264.7 cells were plated at a density of 10,000 cells per well in 96-well plates and incubated for 16 h. Subsequently, the cells were incubated with PGN at varying concentrations (using a multiple dilution method, the maximum concentration was 500 μg/mL) at 37 °C for 24 h. Then, 10 μL of CCK-8 solution was added into each well. After a 1 h incubation, the absorbance at 450 nm was measured with an enzyme meter (Synergy H1, Agilent Technologies, Inc., Santa Clara, CA, USA)

### 4.6. NO and ROS Assays

As recommended by the manufacturer, we used a Nitric Oxide Assay Kit (S0021S, Beyotime Biotech Inc., China) to measure the NO production in the culture supernatant. RAW264.7 cells were plated at a density of 10,000 cells per well in 96-well plates and incubated for 16 h at 37 °C in a 5% CO_2_ atmosphere. After treatment for 12 h, 50 μL of standard or sample was added per well to a 96-well plate, followed by the sequential addition of 50 μL of Griess Reagent I and Reagent II. The absorbance at 540 nm was measured using an enzyme meter (Synergy H1, Agilent Technologies, Inc., USA)

As recommended by the manufacturer, we used a Reactive Oxygen Species Assay Kit with CM-H2DCFDA (S0035S, Beyotime Biotech Inc., China) to measure the production of ROS in the culture supernatant. CM-H2DCFDA was diluted with serum-free culture medium at a ratio of 1:1000 to a final concentration of 5 micromoles per liter. Cells were grown at 37 °C in 5% CO_2_ atmosphere on a 6-well microplate at a density of 2 × 10^5^ cells per well. After 12 h of treatment with PGN, the cells were collected, suspended in diluted CM-H2DCFDA at a concentration of 1 × 10^6^/mL, and incubated in a 37 °C cell culture incubator for 30 min. The suspension was inverted and mixed every 3–5 min to ensure full contact between the probe and the cells. The cells were then washed three times with serum-free cell culture medium to thoroughly remove any CM-H2DCFDA that had not entered the cells. Finally, the cell suspension was transferred to a white 96-well plate, and the fluorescence indicative of ROS was recorded using a plate reader (BioTek Synergy H1, Agilent Technologies, USA)at excitation and emission wavelengths of 480/525 nm.

### 4.7. ELISA, RT-qPCR, and RNA-seq

Cells were cultured at 37 °C in 5% CO_2_ in a 6-well microplate at a density of 2 × 10^5^ cells per well for 16 h. Then, different concentrations of PGN were diluted with fresh medium and added to the cells. The cells were incubated at 37 °C for an additional 12 h, after which the supernatants were collected for tumor necrosis factor-α (TNF-α) and interleukin-1β (IL-1β) assays. The levels of TNF-α and IL-1β in the supernatant were determined using an enzyme-linked immunosorbent assay (ELISA) kit (PT512 and PI301, Beyotime Biotech Inc., China) according to the manufacturer’s recommended protocol.

Total RNA was extracted from different groups of RAW264.7 cells using an RNA extraction kit (DP451, TIANGEN biotech (BEIJING) Co., Ltd., Beijing, China) and reverse transcribed into cDNA by PCR. The specific steps were as follows: Calculate 500 ng total RNA according to the concentration measurement results, add 1 μL of Anchored Oligo (dT)_18_ primer, and add Rnasefree water up to 9 μL for a total volume less than 9 μL. Then, add 10 μL of 2× TS Reaction and 1 μL of Trans Script RT/RI Enzyme to each tube, incubate at 42 °C for 15 min, and inactivate EasyScript RT/RI at 85 °C for 5 s. The primers used are listed in Table 3. The real-time quantitative PCR reaction system is shown in Table 4, and the experimental procedure was as follows: 10 min at 95 °C, 15 s at 95 °C, 1 min at 60 °C, amplification for 40 cycles, and 15 s at 95 °C. Target gene expression was compared between samples by normalizing it to GAPDH expression and using the 2^−ΔΔCt^ formula.

For eukaryotic reference transcriptome analysis, cells were cultured at 37°C in a 5% CO_2_ atmosphere in a 6-well microplate at a density of 2 × 10^5^ cells per well for 16 h. Then, different concentrations of PGN were diluted with fresh medium and added to the cells. The cells were incubated at 37 °C for 12 h. Subsequently, the cells were collected and sent for eukaryotic reference transcriptome analysis by Shanghai Personalbio Technology Co., Ltd. (Shanghai, China)

### 4.8. Statistical Analysis

All experiments were performed in triplicate, and the results were expressed as mean ± standard deviation (SD). The data were statistically analyzed using SPSS statistics software, version 26 (SPSS Inc., Chicago, IL, USA). A one-way ANOVA followed by Duncan’s post-hoc test was used. Results were considered statistically significant when *p* < 0.05.

## 5. Conclusions

In conclusion, our findings confirm that the PGN extracted from *Corynebacterium glutamicum* belongs to the Lys type, with a chemical structure of NAG-(β-1,4)-NAM-l-Ala-d-Glu-l-Lys-d-Ala. The core finding of this study is that PGN can activate the NOD1 receptor, bind to RIP2, and subsequently activate the NF-κB and MAPK signaling pathways, leading to the production of cytokines and reactive oxygen species. Furthermore, it affects the innate immune response and enhances host immunity. Importantly, the application of PGN also provides a new way to utilize amino acid fermentation by-products.

## Figures and Tables

**Table 1:** PGN composition from *Corynebacterium glutamicum*.

Category	Total Carbohydrates	Hexosamine	Protein	Fat
Percentage %	33.54	25.62	29.94	1.9

**Table 2:** MALDI-MS TOF/TOF analysis of PGN from *Corynebacterium glutamicum*.

Amino Acid	(Molar Ratio%)
Glutamic acid (Glu)	4.48
Aspartic acid (Asp)	4.40
Leucine (Leu)	3.78
Lysine (Lys)	3.24
Histidine (His)	2.65
Valine (Val)	2.60
Isoleucine (Ile)	2.57
Alanine (Ala)	2.43

**Table 3:** Specific primers for RT-qPCR analysis.

Gene	Sequence 5′→3′	Accession No.
*TNF-α*	F: GTGCCAGCCGATGGGTTGTAC	NM_013693.3
	R: TGACGGCAGAGAGGAGGTTGAC	
*IL-1β*	F: GCAGCAGCACATCAACAAGAGC	NM_008361.3
	R: AGGTCCACGGGAAAGACACAGG	
*IL-6*	F: GCCTTCTTGGGACTGATGCT	NM_031168.1
	R: GGTCTGTTGGGAGTGGTATCC	
*ERK2*	F: GCAGATCCAGATCATGATCACAC	NM_001038663.1
	R: CTGTGACTGAAGATGGTGACTC	
*iNOS*	F: TCTGCTGGCTTCCTGCTCTCC	NM_010927.3
	R: TCTCCGTGGGCGTGTGATCC	
*COX2*	F: GACAGATTGCTGGCCGGGTTG	NM_011198.5
	R: CAGGGAGAAGCGTTTGCGGTAC	
*NOD2*	F: CAGGTCTCCGAGAGGGTACTG	NM_145857.2
	R: GCTACGGATGAGCCAAATGAAG	
*NOD1*	F: ACAACAGGCGAACTATCTGCGTCA	NM_001171007.1
	R: TCTTAACCGGAAGTAGGCGGAAG	
*RIP2*	F: GGCCATTGTTGAGACCAGATGA	NM_001329751.1
	R: CGTTTCGTAGCCGTGAAGTTTA	
*GAPDH*	F: GTAACCCGTTGAACCCCATT	NM_001289726.1
	R: CCATCCAATCGGTAGTAGCG	
*IKKβ*	F: AGGCGACACGTGAACAGAT	NM_001159774.2NP
	R: CTAAGAGCGGATGCGATG	
*IKKα*	F: GCAGACCGTGAACATCCTCT	NM_001162410.1
	R: TCCAGGACAGTGAACGAGTG	
*MyD88*	F: TGGTGGTGGTTGTTTCTGAC	NM_010851.3
	R: AGTCCTTCTTCATCGCCTTG	

**Table 4:** The reaction system for RT-qPCR analysis.

Reagent	Added (µL)
cDNA	2
Upstream primer	0.6
Downstream primer	0.6
SYBR Green PCR Master Mix	10
Ultrapure water	6.8

## Data Availability

The original contributions presented in the study are included in the article material; further inquiries can be directed to the corresponding author. The raw data on the transcriptome of RAW264.7 cells were deposited in NCBI’s Sequence Read Archive (SRA) database and are accessible through SRA accession number SRP538918.

## References

[1] Martin L.B.B., Kikuchi S., Rejzek M., Owen C., Reed J., Orme A., Misra R.C., El-Demerdash A., Hill L., Hodgson H. (2024). Complete Biosynthesis of the Potent Vaccine Adjuvant QS-21. Nat. Chem. Biol..

[2] Mekonnen D., Mengist H.M., Jin T. (2022). SARS-CoV-2 Subunit Vaccine Adjuvants and Their Signaling Pathways. Expert Rev. Vaccines.

[3] Zhao T., Cai Y., Jiang Y., He X., Wei Y., Yu Y., Tian X. (2023). Vaccine Adjuvants: Mechanisms and Platforms. Signal Transduct. Target. Ther..

[4] Rapaka R.R., Cross A.S., McArthur M.A. (2021). Using Adjuvants to Drive T Cell Responses for Next-Generation Infectious Disease Vaccines. Vaccines.

[5] Xiong B., Chen X., Tu J., Han Z., Meng X., Sun H. (2023). Actinidia Eriantha Polysaccharide Exerts Adjuvant Activity by Targeting Linc-AAM. Int. J. Biol. Macromol..

[6] Ren Z., Luo Y., Meng Z., Zhang J., Yu R., Sun M., Xu T., Li J., Ma Y., Huang Y. (2021). Multi-Walled Carbon Nanotube Polysaccharide Modified Hericium Erinaceus Polysaccharide as an Adjuvant to Extend Immune Responses. Int. J. Biol. Macromol..

[7] Zhao D., Chen X., Wang L., Zhang J., Zhao Z., Yue N., Zhu Y., Fei W., Li X., Tan L. (2023). Bidirectional and Persistent Immunomodulation of Astragalus Polysaccharide as an Adjuvant of Influenza and Recombinant SARS-CoV-2 Vaccine. Int. J. Biol. Macromol..

[8] Wusiman A., Rexiati S., Aziz M., Cheng X., Mai Z., Abulaiti A., Wutikuer A., Rozi P., Abuduwaili A., Abula S. (2022). Preparation and Sulfate Modified of Lagenaria Siceraria (Molina) Standl Polysaccharide and Its Immune-Enhancing Adjuvant Activity. Poult. Sci..

[9] He J., Zhu T., Mao N., Cai G., Gu P., Song Z., Lu X., Yang Y., Wang D. (2024). Cistanche Deserticola Polysaccharide-Functionalized Dendritic Fibrous Nano-Silica as Oral Vaccine Adjuvant Delivery Enhancing Both the Mucosal and Systemic Immunity. Int. J. Biol. Macromol..

[10] Sudo H., Tokunoh N., Tsujii A., Kawashima S., Hayakawa Y., Fukushima H., Takahashi K., Koshizuka T., Inoue N. (2023). The Adjuvant Effect of Bacterium-like Particles Depends on the Route of Administration. Front. Immunol..

[11] Halassy B., Krstanović M., Frkanec R., Tomasić J. (2003). Adjuvant Activity of Peptidoglycan Monomer and Its Metabolic Products. Vaccine.

[12] Vollmer W., Blanot D., De Pedro M.A. (2008). Peptidoglycan Structure and Architecture. FEMS Microbiol. Rev..

[13] Rohs P.D.A., Bernhardt T.G. (2021). Growth and Division of the Peptidoglycan Matrix. Annu. Rev. Microbiol..

[14] Höltje J.-V. (1998). Growth of the Stress-Bearing and Shape-Maintaining Murein Sacculus of Escherichia Coli. Microbiol. Mol. Biol. Rev..

[15] Pasquina-Lemonche L., Burns J., Turner R.D., Kumar S., Tank R., Mullin N., Wilson J.S., Chakrabarti B., Bullough P.A., Foster S.J. (2020). The Architecture of the Gram-Positive Bacterial Cell Wall. Nature.

[16] Egan A.J.F., Errington J., Vollmer W. (2020). Regulation of Peptidoglycan Synthesis and Remodelling. Nat. Rev. Microbiol..

[17] Bastos P.A.D., Wheeler R., Boneca I.G. (2021). Uptake, Recognition and Responses to Peptidoglycan in the Mammalian Host. FEMS Microbiol. Rev..

[18] Khanjani M.H., Sharifinia M., Emerenciano M.G.C. (2023). A Detailed Look at the Impacts of Biofloc on Immunological and Hematological Parameters and Improving Resistance to Diseases. Fish Shellfish Immunol..

[19] Liu X., Xu Z., Chang X., Fang J.K., Song J., He J., Tai Z., Zhu Q., Hu M. (2021). Enhanced immunity and hemocytes proliferation by three immunostimulants in tri-spine horseshoe crab Tachypleus tridentatus. Fish Shellfish Immunol..

[20] Wang L.-S., Zhu H.-M., Zhou D.-Y., Wang Y.-L., Zhang W.-D. (2001). Influence of Whole Peptidoglycan of Bifidobacterium on Cytotoxic Effectors Produced by Mouse Peritoneal Macrophages. World J. Gastroenterol..

[21] Habjanec L., Halassy B., Tomašić J. (2010). Comparative Study of Structurally Related Peptidoglycan Monomer and Muramyl Dipeptide on Humoral IgG Immune Response to Ovalbumin in Mouse. Int. Immunopharmacol..

[22] Ray D., Anand U., Jha N.K., Korzeniewska E., Bontempi E., Proćków J., Dey A. (2022). The Soil Bacterium, *Corynebacterium glutamicum*, from Biosynthesis of Value-Added Products to Bioremediation: A Master of Many Trades. Environ. Res..

[23] Sanchez S., Rodríguez-Sanoja R., Ramos A., Demain A.L. (2018). Our Microbes Not Only Produce Antibiotics, They Also Overproduce Amino Acids. J. Antibiot..

[24] Wu Z., Pan D., Guo Y., Zeng X. (2013). Structure and Anti-Inflammatory Capacity of Peptidoglycan from Lactobacillus Acidophilus in RAW-264.7 Cells. Carbohydr. Polym..

[25] Porfírio S., Carlson R.W., Azadi P. (2019). Elucidating Peptidoglycan Structure: An Analytical Toolset. Trends Microbiol..

[26] Dahiya Y., Pandey R.K., Bhatt K.H., Sodhi A. (2010). Role of Prostaglandin E2 in Peptidoglycan Mediated iNOS Expression in Mouse Peritoneal Macrophages in Vitro. FEBS Lett..

[27] Song X., Li F., Zhang M., Xia Y., Ai L., Wang G. (2022). Effect of D-Ala-Ended Peptidoglycan Precursors on the Immune Regulation of Lactobacillus Plantarum Strains. Front. Immunol..

[28] Liu C., Chu D., Kalantar-Zadeh K., George J., Young H.A., Liu G. (2021). Cytokines: From Clinical Significance to Quantification. Adv. Sci..

[29] Hamann L., El-Samalouti V., Ulmer A.J., Flad H.D., Rietschel E.T. (1998). Components of Gut Bacteria as Immunomodulators. Int. J. Food Microbiol..

[30] Kolling Y., Salva S., Villena J., Marranzino G., Alvarez S. (2015). Non-Viable Immunobiotic Lactobacillus Rhamnosus CRL1505 and Its Peptidoglycan Improve Systemic and Respiratory Innate Immune Response during Recovery of Immunocompromised-Malnourished Mice. Int. Immunopharmacol..

[31] Sun Q., Liu X., Li X. (2022). Peptidoglycan-Based Immunomodulation. Appl. Microbiol. Biotechnol..

[32] Bersch K.L., DeMeester K.E., Zagani R., Chen S., Wodzanowski K.A., Liu S., Mashayekh S., Reinecker H.-C., Grimes C.L. (2021). Bacterial Peptidoglycan Fragments Differentially Regulate Innate Immune Signaling. ACS Cent. Sci..

[33] Wolf A.J., Underhill D.M. (2018). Peptidoglycan Recognition by the Innate Immune System. Nat. Rev. Immunol..

[34] Sekine K., Toida T., Saito M., Kuboyama M., Kawashima T., Hashimoto Y. (1985). A new morphologically characterized cell wall preparation (whole peptidoglycan) from Bifidobacterium infantis with a higher efficacy on the regression of an established tumor in mice. Cancer Res..

[35] Zhang W.-H., Wu J., Weng L., Zhang H., Zhang J., Wu A. (2020). An Improved Phenol-Sulfuric Acid Method for the Determination of Carbohydrates in the Presence of Persulfate. Carbohydr. Polym..

[36] Bradford M.M. (1976). A Rapid and Sensitive Method for the Quantitation of Microgram Quantities of Protein Utilizing the Principle of Protein-Dye Binding. Anal. Biochem..

[37] Leaback D.H., Walker P.G. (1963). On the Morgan-Elson Reaction. Biochim. Biophys Acta.

